# Body representation disturbances in visual perception and affordance perception persist in eating disorder patients after completing treatment

**DOI:** 10.1038/s41598-017-16362-w

**Published:** 2017-11-23

**Authors:** Manja M. Engel, Anouk Keizer

**Affiliations:** 1 Utrecht University, Faculty of Social and Behavioural Sciences, Experimental Psychology/Helmholtz Institute, PO Box 80.140, 3508TC Utrecht, The Netherlands; 2 Leontienhuis, Zuidplasweg 13, 2761JK Zevenhuizen, The Netherlands

## Abstract

Body image disturbances (BID) are a key feature of eating disorders (ED). Clinical experience shows that BID exists in patients who *Completed their Eating Disorder Treatment* (CEDT), however studies concerning BID in CEDT patients are often limited to cognition and affect, measured by interviews and questionnaires. The current study is the first systematic study investigating the full scope of the mental body representation, including bodily attitudes, visual perception of body size, tactile perception, and affordance perception in CEDT patients. ED patients (*N* = 22), CEDT patients (*N* = 39) and healthy controls (HC; *N* = 30) were compared on BID tasks including the Body Attitude Test (BAT), Visual Size Estimation (VSE), Tactile Estimation Task (TET), and Hoop Task (HT). Results on the BAT show higher scores for ED patients compared to CEDT patients and HC but no difference between CEDT patients and HC. Both ED and CEDT patients show larger overestimations on the VSE and HT compared to HC, where ED patients show the largest overestimations. No group differences were found on the TET. The results indicate the existence of disturbances in visual perception and affordance perception in CEDT patients. Research focussing on more effective treatments for ED addressing multiple (sensory) modalities is advised.

## Introduction

An eating disorder (ED) is a severe mental illness that causes impairments in psychosocial functioning and/or physical health^1^. Patients diagnosed with anorexia nervosa (AN), bulimia nervosa (BN) and Other Specified Feeding or Eating Disorders (OFSED) often report to suffer from an unbearable feeling of being too fat, despite having a healthy body weight or being (severely) underweight. This incorrect notion of one’s own body size or shape is called *body image disturbance* (BID) and found to be a key feature in AN, BN and OFSED e.g.^2–7^. BID is strongly associated with the development and maintenance of an eating disorder e.g.^8–10^. Moreover, relapse is predicted by the severity of BID^11–13^. It is therefore plausible that BID complicates the recovery process of ED patients. BID has already been investigated in ED patients e.g.^14–16^. To ensure fewer relapses in ED patients and optimize ED treatment, it is important to acquire a clearer understanding of body image in patients that *Completed their Eating Disorder Treatment* (CEDT). The present study is the first systematic study focussing on aspects of BID in CEDT patients in order to provide insight into which aspects of BID might still be affected after recovery.

This study follows a cognitive neuroscience perspective on BID, in which it is assumed that the brain processes primarily multimodal information pertaining the body from different sensory modalities and integrates it into an coherent and abstract higher order representation of the body see e.g.^17–23^. The mental body representation stores information concerning the body, including the dimensions of the body size. Terminology of BID refers to mental body image, emphasising the visual aspects of the mental body representation. However in this paper we use the term BID in a broader sense referring to the full scope of the mental body representation, containing visual, tactile, cognitions, affect, etc. For this study we focussed on specific information regarding the size of the mental body representation.

Studies on BID in ED patients have found disturbances in the bodily attitudes (thinking and/or imagining oneself as fat), visual perception of the body (seeing oneself as fat)^15,16,24^, haptic perception^25^, tactile perception (feeling touch on the skin)^26–28^, and affordance perception/bodily action (knowing what one can do with one’s body given its size)^29,30^. These disturbances are suggested to be linked to an enlarged mental representation of body size^23,26,31^.

Taken together, BID in ED are complex and disturbances are found in bodily attitudes and in multiple sensory modalities. However, the existing interventions are mostly aimed at improving cognition and visual perception, and they seldom include all sensory modalities. Treatment for ED is mainly based on Cognitive Behavioural Therapy (CBT) models primarily addressing, identifying and challenging weight and shape related thoughts e.g.^32–34^. Body image interventions, such as mirror exposure e.g.^35^, are implemented in evidence-based CBT for ED. Furthermore, no standardized intervention model for BID in the context of ED exists, causing a lot of heterogeneity amongst BID treatment approaches e.g.^24,36,37^. Given the severity and long recovery process of BID, an effective treatment is of importance.

Recovery is often determined based on BMI, self-report and questionnaires^38,39^. It is striking that (recovery of) BID is mainly assessed with self-report measures such as questionnaires measuring cognition and affect regarding the body and its size e.g.^40,41^. BID in ED is more than disturbance in bodily cognition and affect, and also expressed in other levels of body representation, such as tactile perception and affordance perception/bodily action, that cannot be assessed using only self-report measures e.g.^2,14,23,42^.

There are some indications that BID persists after recovery^43–45^. Eshkevari and colleagues^43^ found a disturbance in the integration of visual and tactile information in ED patients and recovered individuals. They concluded that a disturbance in the experience of the bodily self in people with an eating disorder remains to a large degree following weight regain and is a suggestive trait factor. A trait factor refers to a stable pattern of behaviour, thoughts and emotions over a long period of time. This again shows that BID is a complex aspect of an ED and does not simply diminish following weight gain. Although at this point little research towards BID after treatment has been conducted, clinical experience shows that patients continue to struggle with BID, even after otherwise successful treatment.

In the present study we systematically investigated the mental body representation in patients who completed ED treatment (CEDT group), and compared them to acute ED patients (ED group) and healthy controls (HC group). We specifically assessed four domains in which BID in ED patients are found: bodily attitudes, visual perception of body size, tactile perception, and affordance perception. From previous studies we know that ED patients hold negative attitudes towards their own body^10,46–49^, therefore we expect to find stronger negative bodily attitudes in ED patients compared to CEDT patients and HC. Considering the current treatment focus, where ED patients learn new cognitive coping mechanisms to deal with feeling fat, we expect no differences in bodily attitudes between CEDT patients and HC. To measure the existence of (multi)sensory BID symptoms in CEDT patients, ED patients and HC, we included a Visual Size Estimation task (VSE)^30^, previously used by e.g.^14,50–52^, Tactile Estimation Task (TET)^26,27^, and the Hoop Task (HT)^53^. In accordance with previous findings of mental body representation in ED and the known persistence of body image disturbance in ED^43,44^, we hypothesize that ED patients and CEDT patients will show larger size estimations on the VSE, TET, and HT compared to HC. No differences are expected between ED and CEDT patients on these tasks.

## Methods

### Ethics statement

The current study was approved by the ethics committee of the Utrecht University, Faculty of Social and Behavioural Sciences, Experimental Psychology. The study adhered to the tenets of the Declaration of Helsinki (2013). Each participant was informed about the study; they received oral and written information on the purpose and procedure prior to the experiment. The participants all signed an informed consent form before taking part in the study.

### Participants

ED patients and CEDT patients were recruited from the Leontienhuis, Stichting JIJ, and treatment centre GGZ Rivierduinen Eetstoornissen Ursula, all located in the Netherlands. The Leontienhuis and Stichting JIJ are institutions that aim to improve the quality of life of people with an eating disorder, their family and those involved. The Leontienhuis is a non-treatment institution where help and support is provided by people recovered from an eating disorder. For more information see www.leontienhuis.nl and www.stichting-jij.nl. HC were volunteers from the Leontienhuis, and undergraduate students who were recruited from Utrecht University. A total of 91 females participated in this study: 22 ED patients, 39 CEDT patients, and 30 HC (9 from the Leontienhuis and 21 from the Utrecht University).

None of the ED patients were hospitalized during the testing phase of this study. All patients were provided with treatment according to the national guidelines for care for ED in The Netherlands, which mainly concerns CBT. ED patients received care at different locations in The Netherlands. ED patients and CEDT patients were included who were diagnosed by a psychiatrist or psychologist. One ED patient and three CEDT patients were not assessed by a psychiatrist or psychologist during their ED and did not receive a formal diagnosis. These participants were orally interviewed, and their symptoms matched DSM-V criteria for ED. See Table 1 for the clinical assessment of the ED patients and CEDT patients, note that diagnosis information was missing for two CEDT patients. CEDT patients were included when they completed their ED treatment and orally reported to be fully recovered during time of testing. All ED patients received treatment during time of testing. Inclusion criteria for HC were ‘no presence of ED symptoms in past and present’.

**Table 1:** Demographics and clinical assessment of ED, CEDT and HC.

	ED (*N* = 22)	CEDT (*N* = 39)	HC (*N* = 30)
**Demographics**			
Age, M (SD)	32.00 (10.25)	31.74 (10.74)	30.20 (14.22)
BMI (kg/m^2^), M (SD)	19.29 (2.00)	20.47 (2.14)	22.80 (3.79)
Right-handedness, N (%)	18 (81.8%)	34 (87.2%)	29 (96.7%)
Past pregnancy, N (%)	8 (36.4%)	12 (30.8%)	6 (20.0%)
**Diagnoses**	*N* (%)	*N* (%)	
AN	13 (59.1%)	19 (48.7%)	—
BN	—	3 (7.7%)	—
OFSED	3 (13.6%)	3 (7.7%)	—
AN&BN	1 (4.5%)	4 (10.3%)	—
AN&OFSED	4 (18.2%)	5 (12.8%)	—
No diagnoses	1 (4.5%)	3 (7.7%)	—
	*M* (*SD*)	*M* (*SD*)	
Age ED diagnosis in yrs	25.17 (10.28)	18.95 (6.71)	—
Duration ED in yrs	9.36 (9.18)	7.52 (5.72)	—
Duration treatment ED in yrs	3.32 (3.26)	2.50 (2.37)	—
Number of relapses	3.08 (3.90)	2.05 (1.80)	—
Time since completion ED treatment in yrs	—	5.78 (5.45)	—

We checked for correlations of all BID measurements with ‘age ED diagnoses’, ‘duration ED’, ‘duration ED treatment’, ‘number of relapses’, and ‘time since completion ED treatment’, as described in Table 1. There were some significant correlations but they did not survive the Bonferroni correction (critical *p* = 0.003; all *p*’s > 0.030) and were therefore not used as covariates in analyses.

#### Demographics

In a demographic questionnaire age, weight, handedness, education, and past pregnancy were assessed. Height was measured by the examiner. Means and standard deviations by group are given in Table 1. No significant age differences were found between groups, *F*(2, 87) = 0.19, *p* = 0.826. BMI was significantly different between groups, *Welch’s F* (2, 8.03) = 9.47, *p* < 0.001, ω = 0.42. Post-hoc analysis using the Games-Howell correction showed that BMI in HC was significantly higher than in ED patients (*p* = 0.001) and CEDT patients (*p* < 0.05). No differences in BMI were found between ED patients and CEDT patients (*p* = 0.145). Mean level of education was secondary vocational education for ED patients and CEDT patients and high school for HC. Note that the majority of the HC were undergraduate students, in the process of completing their higher education. We checked for correlations between all BID measurements and self-reported handedness, education and past pregnancy, as rapid changes in body size during that period might affect body image. No significant relations were found, therefore these variables were not used as covariates in analyses (all *p*’s ≥ 0.169).

### Materials and procedure

After the signing of the informed consent, patients filled out a questionnaire with questions concerning demographic and clinical information and proceeded with the tasks in order of which they are described below.

#### Body Attitude Test (BAT)

Body attitude was assessed with the Dutch version of the BAT, Lichaams Attitude Vragenlijst (LAV)^54^. This Dutch questionnaire was specifically developed for patients with AN and BN, and is reported to be a valid and reliable instrument to measure the subjective attitude of the body. The BAT consists of 20 self-report items (e.g. ‘*I think I am fat*.’) with a 6-point Likert scale (1 = never, to 6 = always). The items are divided into three subscales: ‘negative appreciation of body size’, ‘lack of familiarity with one’s own body’, and ‘general dissatisfaction’. A higher score represents a more negative body attitude.

#### Visual Size Estimation Task (VSE)

The VSE^30^ was used to measure visual body perception. Points of interest were shoulders, waist, and hips. Standing in front of the wall with a distance of approximately one meter, participants estimated their width of each body part by placing two arrow shaped stickers horizontally on the wall. The space between the stickers indicated the estimated width. Stickers were removed before the next estimation to prevent participants from comparing estimates. The order of estimations was counterbalanced over participants. At the end of the experiment, the actual size of the shoulders, waist and hips of the participant was measured by the experimenter. The percentage of misestimation was calculated by: 100*((body size estimation − actual body size)/body size estimation), where a higher percentage indicates a larger overestimation.

#### Tactile estimation task (TET)

**Figure 1:**
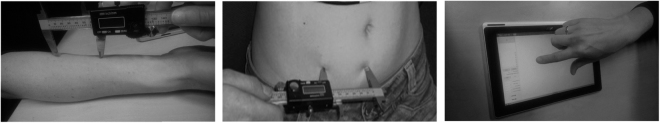
Tactile Estimation Task. Tactile distances were presented by a caliper on the right arm as shown in the left photo. The center photo shows the abdomen condition. Estimations were made by placing their index finger and thumb on a tablet as shown in the photo on the right.

 A shortened version of the TET used by^26^ was used to measure perception of tactile distances of the right forearm (emotionally neutral body part) and right side of the abdomen (emotionally salient body part). The order of the arm and abdomen was counterbalanced over participants. Tactile stimuli were presented with a caliper with distances of 50, 60 and 70 mm in a randomized order over three trials, with a total of nine trials. The estimation of the width of the stimuli was made with the index finger and thumb of the right hand, by placing these fingers on a tablet, see Fig. 1. To ensure no visual interference during the presentation and estimation of tactile stimuli, participants were asked to close their eyes. Mean distances in mm were calculated for both the arm and abdomen condition for each group.

#### Hoop Task (HT)

The HT as designed by^53^ was used for body-scaled action measurements. Participants were asked to judge whether their body would fit through a hoop, and step through the hoop if they thought it would fit. Fifteen hoops were presented one by one in randomized order to the participant. Hoops were placed on the floor with a distance of approximately one meter from the participant. If the hoop was judged by the participant to be big enough, the participant had to step inside the hoop and lift it over her head. The hoops were made from PVC pipes and were slightly mouldable. The hoops differed in sizes with diameter ranging from 24 to 52 cm, with 2 cm increments. Participants were asked to look away when a new hoop was selected and presented, to prevent the participant from directly comparing hoops. At the end of all tasks the actual hoop size was measured by asking the participant to step through hoops until the smallest one was found. With the smallest hoop estimate and the actual hoop size, a percentage of misestimation of hoop diameter in cm was calculated for each participant (100*(hoop size estimations − actual hoop size)/actual hoop size), where higher positive percentages indicate higher overestimations.

### Data Availability

The datasets generated during and/or analyzed during the current study are available from the corresponding author on reasonable request.

## Results

### Body Attitude Test

One participant (*N*_CEDT_ = 1) was not able to participate in the BAT. This participant was excluded from data analysis for this task. Results of the ANOVA showed significant differences between groups on the total BAT score, *Welch’s F*(2, 46.07) = 55.27, *p* < 0.001, ω = 0.70, and on BAT subscales: ‘negative appreciation with one’s body size’, *Welch’s F*(2, 50.44) = 37.87, *p* < 0.001, ω = 0.23; ‘lack of familiarity with one’s own body’, *Welch’s F*(2, 43.10) = 38.74, *p* < 0.001, ω = 0.67; ‘general dissatisfaction with one’s own body’, *Welch’s F*(2, 55.74) = 59.14, *p* < 0.001, ω = 0.66. See Table 2 for means and standard deviations.

**Table 2:** Means and standard deviations of the subscales and total scores of the BAT by group.

	ED (*N* = 21)		CEDT (*N* = 38)		HC (*N* = 30)	
	*M*	*SD*	*M*	*SD*	*M*	*SD*
BAT Total	64.23	16.76	31.11	19.72	23.07	8.42
BAT Negative appreciation	25.45	8.23	9.53	9.09	7.90	5.74
BAT Lack of familiarity	20.95	8.72	9.74	7.53	4.60	3.10
BAT General dissatisfaction	14.91	2.98	6.92	5.03	6.07	3.08

Planned contrasts were used to further examine group differences. ED patients had significant higher total BAT scores compared to CEDT patients and HC, *t*(31.86) = 9.31, *p* < 0.001 (1-tailed), *r* = 0.86. No difference was found between CEDT patients and HC (*t*(52.50) = 2.27, *p* = 0.140 (1-tailed)). ED patients had significant higher scores than CEDT patients and HC on BAT subscales ‘negative appreciation with one’s body size’, *t*(87) = 8.62, *p* < 0.001 (1-tailed), *r* = 0.68; ‘lack of familiarity with one’s own body’, *t*(26.68) = 6.97, *p* < 0.001 (1-tailed), *r* = 0.80; ‘general dissatisfaction with one’s own body’, *t*(48.44) = 10.45, *p* < 0.001 (1-tailed), *r* = 0.83. Scores between CEDT patients and HC showed a significant difference on the BAT subscale ‘lack of familiarity with one’s own body’, *t*(51.59) = 3.82, *p* < 0.001 (1-tailed), *r* = 0.47. No significant difference was found between CEDT patients and HC on the BAT subscale ‘negative appreciation with one’s body size’, *t*(87) = 0.84, *p* = 0.201 (1-tailed), and BAT subscale ‘general dissatisfaction with one’s own body’, *t*(62.53) = 0.86, *p* < 0.131 (1-tailed).

Using the BAT as an indication for bodily attitudes, these results show that ED patients held stronger negative attitudes towards their body compared to CEDT patients and HC. CEDT patients and HC did not differ in body attitude except for ‘lack of familiarity with one’s own body’ with a more negative body attitude in CEDT patients compared to HC.

### Visual size estimation

One participant (*N*_CEDT_ = 1) did not participate in the VSE as she indicated that the task was too anxiety-provoking, this participant was excluded from data analysis for this task. Estimations of size of shoulder, waist, and hips were compared between groups. An ANOVA showed between-group differences for shoulders, *Welch’s F*(2, 45.47) = 4.10, *p* < 0.05, ω = 0.26, (*M*_ED_ = 12.32, *SD* = 27.09; *M*_CEDT_ = 4.54, *SD* = 18.04; *M*_HC_ = −4.12, *SD* = 15.61); waist, *F*(2, 86) = 4.93, *p* < 0.05, ω = 0.28, (*M*_ED_ = 31.69, *SD* = 35.52; *M*_CEDT_ = 11.57, *SD* = 22.45; *M*_HC_ = 9.25, *SD* = 25.95); and hips, *Welch’s F*(2, 44.41) = 4.31, *p* < 0.05, ω = 0.33, (*M*_ED_ = 26.41, *SD* = 35.03; *M*_CEDT_ = 8.53, *SD* = 21.56; *M*_HC_ = 2.85, *SD* = 16.03.

**Figure 2:**
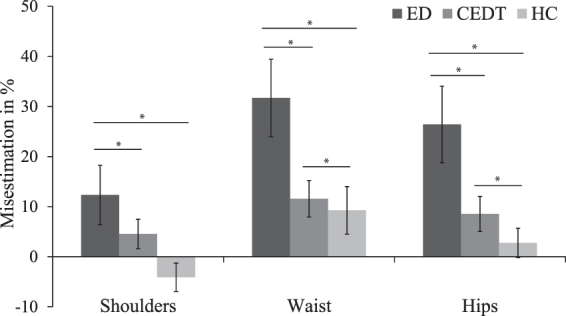
Percentage of misestimations of shoulder-, waist-, and hip- width by ED patients, CEDT patients and HC. Error bars depict S.D. *p < 0.05.

Planned contrast analysis revealed that both ED patients and CEDT patients had a higher percentage of misestimation of shoulder width compared to HC, *t*(58.08) = 2.88, *p* < 0.05 (1-tailed), *r* = 0.36. Estimations of shoulder width between ED patients and CEDT patients showed no significant difference (*t*(30.03) = 1.18, *p* = 0.124 (1-tailed)). Size estimation of width of waist showed higher misestimations in ED patients and CEDT patients compared to HC, *t*(86) = 2.00, *p* < 0.05 (1-tailed), *r* = 0.21. ED patients and CEDT patients also showed significant difference in waist size estimations, with a higher percentage of misestimation in ED patients, *t*(86) = 2.73, *p* < 0.05 (1-tailed), *r* = 0.28. Size estimation of width of hips showed a higher percentage of misestimation in ED patients and CEDT patients compared to HC, *t*(51.15) = 2.87, *p* < 0.05 (1-tailed), *r* = 0.37. ED patients also showed a higher percentage of misestimation than CEDT patients, *t*(28.58) = 2.13, *p* < 0.05 (1-tailed), *r* = 0.37, see Fig. 2.

Taken together, the results from the VSE showed differences in estimations of body size per group, where ED patients had the highest misestimations followed by CEDT patients and then HC.

### Tactile Estimation Task

Four participants (*N*_ED_ = 2, *N*_CEDT_ = 2) did not participate in both TET arm and TET abdomen as they found the task too anxiety provoking. These participants were therefore excluded from data analysis for this task. TET performances between ED patients, CEDT patients, and HC were compared using a 2 × 3 repeated measures ANOVA with body part (arm and abdomen) versus distance (50 mm, 60 mm, 70 mm). Means and standard deviations are shown in Table 3.

**Table 3:** Means and standard deviations of TET estimates in mm of participants.

	ED (*N* = 20)		CEDT (*N* = 37)		HC (*N* = 30)	
	*M*	*SD*	*M*	*SD*	*M*	*SD*
Total	65.88	18.42	60.92	13.86	64.41	9.84
Arm	67.87	19.34	67.53	16.59	69.33	11.15
Abdomen	66.35	24.50	56.91	14.02	59.49	12.19

Main effects were found for distance, *F*(2, 84) = 109.08, *p* < 0. 001, ω = 0.70 and body part, *F*(2,168) = 8.04, *p* < 0.001, ω = 0.89. There was no significant main effect for group (*F*(2, 84) = 0.87, *p* = 0.419) and no significant interaction for distance*group (*F*(4, 84) = 0.22, *p* = 0.928), indicating that the linear increase in size estimations was independent of group membership. Therefore, tactile distances were averaged in each group with 60 mm as perfect mean estimation. The interaction between body*group was not significant (*F*(2, 84) = 1.88, *p* = 0.159). These results show no difference in tactile estimation on arm and abdomen between ED patients, CEDT patients and HC.

### Hoop task

Two participants (*N*_ED_ = 2) thought they were unable to fit through any of the presented hoops and were therefore excluded from analysis for this task. One participant (*N*_HC_ = 1) was excluded due to prior knowledge of the hoop task. The smallest hoop participants actually fitted through was compared between ED patients, CEDT patients, and HC. There was no significant difference in actual hoop diameter between groups (*F*(2, 87) = 2.58, *p* = 0.081, (*M*_ED_ = 31.36, *SD* = 2.34; *M*_CEDT_ = 31.18, *SD* = 2.50; *M*_HC_ = 32.41, *SD* = 1.96).

**Figure 3:**
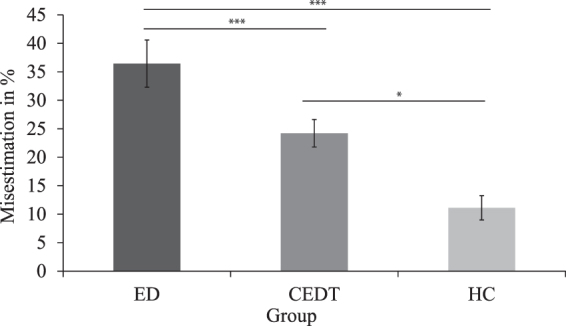
Percentage of hoop size overestimation in ED patients, CEDT patients and HC. Error bars depict S.D. ***p < 0.001, *p < 0.05.

An ANOVA showed significant differences between the groups for estimation of hoop size, *Welch’s F*(2, 44.79) = 18.21, *p* < 0.001, ω = 0.52, (*M*_ED_ = 36.44, *SD* = 18.57; *M*_CEDT_ = 24.20, *SD* = 15.07; *M*_HC_ = 11.13, *SD* = 11.06. Planned contrasts showed that ED patients and CEDT patients had larger percentage of overestimations compared to HC, *t*(38.37) = 5.61, *p* < 0.001 (1-tailed), *r* = 0.67, and ED patients had larger percentage of overestimations than CEDT patients, *t*(32.16) = 2.55, *p* < 0.05 (1-tailed), *r* = 0.41. Taken together these results indicate that ED patients and CEDT patients overestimate their hoop diameter compared to HC where ED patients have the highest overestimations compared to CEDT patients, see Fig. 3.

## Discussion

The current study is the first systematic study that aimed to investigate whether multiple aspects of BID that are found in ED patients see e.g.^14,15,55,56^ may continue to exist after treatment completion for ED since current treatments focuses mainly on disturbed thoughts and emotions, and to a lesser extent on sensory perception e.g.^32–34^. We assessed BID in four different domains: bodily attitudes, visual perception of body size, tactile perception, and affordance perception. BID was measured with tasks previously used by Keizer and colleagues^31^ in studies to BID with ED patients. It was hypothesized that ED patients would have the strongest negative bodily attitudes compared to CEDT and HC, while CEDT patients would show bodily attitudes comparable to HC due to learned coping strategies in ED treatment. We further expected that ED patients and CEDT patients would overestimate their body size in all perceptual modalities compared to HC.

Results of this study confirm our expectations on the existence of BID in CEDT patients in the visual perception and affordance perception domain, whilst being absent in the bodily attitudes. Results regarding bodily attitudes and BID revealed that ED patients hold stronger negative bodily attitudes compared to CEDT patients and HC. As expected, no differences were found in bodily attitudes between CEDT patients and HC. Both ED patient and CEDT patients show larger overestimations of their body size in the visual perception and affordance perception domain compared to HC, where ED patients show the largest overestimations and CEDT are intermediate between HC and ED. In contrast to expectations, no differences in tactile perception were found between ED patients, CEDT patients and HC. Results of this study clearly show the striking existence of BID symptoms after patients completed their eating disorder treatment. In addition to existing scientific knowledge of severity of BID in relation to development, maintenance and relapse in ED^8–13^, the DSM-V clearly states BID as a key symptom of AN and OFSED. This new evidence of an existing symptom of a disease after treatment completion denounces the degree of recovery, especially in a complex and severe disorder. Regarding the results of this systematic study on BID, it seems prudent to consider more effective body image interventions.

The present study is not free of limitations that should be taken into account in future research. First, one could argue that self-reporting weight is a limitation of the current study. In our previous work weight was always measured by the experimenter. However, we never found a correlation between weight or BMI and the measures of body image, even though the same or similar tasks were used. It appears that perceived body size and related disturbances are independent of current weight or BMI status (see e.g.^26,27,30,31^). The current study does not directly offer an explanation about the absence of significant group differences on the tactile estimation task. Although ED patients show a tendency for an overestimation of tactile distances presented on the abdomen, the results are not conclusive. The current findings are not in accordance with previously reported findings^26–28^ where tactile distances were overestimated by ED patients compared to HC. It should be noted that fewer tactile estimations trials were used in this study compared with the study of Keizer, *et al*.^26^. Perhaps this could account for the absence of significant results, although further research on tactile estimation in CEDT patients is necessary to draw conclusions.

Nevertheless it is evident that BID symptoms are not fully targeted during ED treatment since BID symptoms still exist in CEDT patients. The absence of strong negative bodily attitudes in CEDT patients could substantiate our theory on the effect of the current therapy focus, where patients learn to recognise dysfunctional thoughts and new strategies are taught to cope with these cognitions and negative affect^57^, while too little attention is spent on other BID symptoms such as disturbances in visual body perception and affordance perception.

We believe that the outcome of this study has clear implications for treatment for ED especially in relation to BID. The significance of sensory domains in relation to body image should be recognized and integrated in standardized ED treatments, aiming to adjust the enlarged mental body representation in ED patients to its actual size. Studies that used the Rubber Hand Illusion (RHI)^58^ already showed that it is possible to change the internal body representation when sensory modalities are targeted. During the RHI, a rubber hand and the patients hand are stroked at the same time while only the rubber hand is shown to the participant. After the RHI ED patients estimated the size of their own hand more accurate than before the experiment where they overestimated their hand size^31,59^. Keizer, *et al*.^60^ showed the same effect in a full body illusion in virtual reality, where patients had a more accurate estimation of their body size after the experiment. Note that the effect was found in an experimental setting. Although these are the outcomes of small studies, it is evident that the body representation can be altered. The malleable body representation in ED patients is a positive indicator for BID treatment; engaging multiple sensory domains in BID treatment can probably improve efficiency and effectivity of conventional treatments. More and more researchers investigate the role of bodily experiences in psychiatric populations (e.g. in schizophrenia^61^ and borderline personality disorder^62^). Body representation is a multifaceted concept, and as such can be assessed in several ways. The current findings may serve as an inspiration for different fields, as it shows the importance of systematically assessing different aspects of body representation.

In sum, BID symptoms are found to be present in CEDT patients, indicating that patients are not fully recovered after treatment is finished. This finding is an urgent factor that needs to be addressed since BID is a strong predictor for relapse in ED^11–13^. Distortions are found in visual perception and action/performance modality. Bodily attitudes in CEDT patients do not differ from HC. These results perhaps reflect the limited approach of current treatment methods, focussing mainly on the attitudinal aspect of BID, while a body of literature shows that BID is multifaceted see e.g.^17–23^. Given the current results on the existence of BID in ED patients who have completed treatment, knowledge of severity of BID, combined with the findings of alternation of the body image using a multimodal approach, research to more effective treatments addressing multiple (sensory) modalities in ED is advised.
